# Transcriptome-Enabled Network Inference Revealed the *GmCOL1* Feed-Forward Loop and Its Roles in Photoperiodic Flowering of Soybean

**DOI:** 10.3389/fpls.2019.01221

**Published:** 2019-11-08

**Authors:** Faqiang Wu, Xiaohan Kang, Minglei Wang, Waseem Haider, William B. Price, Bruce Hajek, Yoshie Hanzawa

**Affiliations:** ^1^Department of Biology, California State University, Northridge, CA, United States; ^2^Department of Electrical Computer Engineering, University of Illinois at Urbana-Champaign, Champaign, IL, United States; ^3^Department of Crop Sciences, University of Illinois at Urbana-Champaign, Champaign, IL, United States; ^4^Department of Biosciences, COMSATS University Islamabad, Pakistan

**Keywords:** photoperiodic flowering, transcriptome, network inference, feedforward loop, *Glycine max*

## Abstract

Photoperiodic flowering, a plant response to seasonal photoperiod changes in the control of reproductive transition, is an important agronomic trait that has been a central target of crop domestication and modern breeding programs. However, our understanding about the molecular mechanisms of photoperiodic flowering regulation in crop species is lagging behind. To better understand the regulatory gene networks controlling photoperiodic flowering of soybeans, we elucidated global gene expression patterns under different photoperiod regimes using the near isogenic lines (NILs) of maturity loci (*E* loci). Transcriptome signatures identified the unique roles of the *E* loci in photoperiodic flowering and a set of genes controlled by these loci. To elucidate the regulatory gene networks underlying photoperiodic flowering regulation, we developed the network inference algorithmic package CausNet that integrates sparse linear regression and Granger causality heuristics, with Gaussian approximation of bootstrapping to provide reliability scores for predicted regulatory interactions. Using the transcriptome data, CausNet inferred regulatory interactions among soybean flowering genes. Published reports in the literature provided empirical verification for several of CausNet's inferred regulatory interactions. We further confirmed the inferred regulatory roles of the flowering suppressors *GmCOL1a* and *GmCOL1b* using *GmCOL1* RNAi transgenic soybean plants. Combinations of the alleles of *GmCOL1* and the major maturity locus *E1* demonstrated positive interaction between these genes, leading to enhanced suppression of flowering transition. Our work provides novel insights and testable hypotheses in the complex molecular mechanisms of photoperiodic flowering control in soybean and lays a framework for *de novo* prediction of biological networks controlling important agronomic traits in crops.

## Introduction

The flowering gene network controls the transition from vegetative to reproductive growth, a major life cycle event that determines reproductive success and productivity of plants. The flowering gene network in the model plant *Arabidopsis thaliana* (Arabidopsis) is one of the most well-studied biological networks, consisting of multiple subnetworks that process perception and signaling of different exogenous and endogenous signals (Andres and Coupland, 2012; Wu and Hanzawa, 2014). However, our knowledge about the molecular bases of plant diversity in flowering response to environments remains limited, especially in economically important crop species.

The photoperiodic flowering pathway, a subnetwork of the flowering gene network, provides a point of comparison across species that possess diverse photoperiodic response in flowering regulation (Wu and Hanzawa, 2014). *GIGANTEA* (*GI*) and *CONSTANS* (*CO*) are known conserved genes among distantly related flowering plants that play central roles in photoperiodic flowering control (Song et al., 2015; Brambilla et al., 2017). In the facultative long-day flowering plant Arabidopsis, GI is a component of the circadian clock and a regulator of the nuclear protein CO. GI forms a complex with FLAVIN-BINDING, KELCH REPEAT, F-BOX 1 (FKF1) in the late afternoon under long day and triggers degradation of CYCLING DOF FACTOR 1 (CDF1) and its homologs that repress *CO* mRNA expression, resulting in upregulation of *CO* transcripts, at the end of the day (Sawa et al., 2007). The photoreceptors PHYTOCHROME A (PHYA), CRYPTOCHROME 1 (CRY1), and CRY2, as well as FKF1, stabilize CO protein toward the end of the day, leading to activation of the flowering promoter *FLOWERING LOCUS T* (*FT*) that induces floral meristem identity genes, thereby initiating flowering (Valverde et al., 2004; Song et al., 2012). In addition to this GI-CO pathway, GI is known to induce *FT* through two CO-independent mechanisms. GI activates the small non-coding RNA *microRNA172* (*miR172*), which induces *FT* through repression of APETALA2 (AP2)-like transcription factors including *RELATED TO AP2.7* (*RAP2.7*)/*TARGET OF EARLY ACTIVATION TAGGED 1* (*TOE1*) that function as flowering inhibitors (Jung et al., 2007). In addition, GI also binds directly to the *FT* promoter and activates *FT* (Sawa and Kay, 2011).

The central mechanisms of photoperiodic flowering appear conserved in the short-day flowering plant rice (*Oryza sativa*) (Tsuji et al., 2011; Brambilla et al., 2017). As in Arabidopsis, the rice GI ortholog OsGI activates the *CO* ortholog *Heading date1* (*Hd1*) under flowering-inductive short day (Yano et al., 2000). Hd1 then activates expression of the *FT* homologs *Hd3a* and *RFT1* (Komiya et al., 2008). Rice also possesses unique variations and novel components in photoperiodic flowering control. Unlike Arabidopsis CO, rice Hd1 plays a dual role in flowering regulation: a promoter of *Hd3a* under short day and an inhibitor under long day. In addition, rice utilizes the unique component *Early heading date1* (*Ehd1*) encoding a B-type response regulator (Doi et al., 2004). *Ehd1* is induced by OsGI and acts as a flowering promoter under both short day and long day through activation of different *FT* homologs: *Hd3a* under short day and *RFT1* under long day.

The short-day flowering crop soybean (*Glycine max*) provides insightful comparisons with Arabidopsis and rice. Recent efforts demonstrate that the key players of the flowering gene network are well conserved in soybean (Cao et al., 2017). A homolog of *GI*, *GmGIa*, has been isolated as the causal gene of the major maturity-controlling locus *E2*, and the homologs of the photoreceptor *PHYA* as the causal genes of *E3* and *E4*. In addition, the conserved roles of the soybean *FT* homologs *GmFT2a* and *GmFT5a* in flowering induction have been reported, as well as *FT2c* in wild soybean *Glycine soja* (Wu et al., 2017). The roles and regulation of *microRNA*s also appear to be generally conserved in soybean (Cao et al., 2017). *E2*/*GIa* is shown to promote maturation of *GmmiR172a* that cleaves the flowering inhibitor *GmTOE4a*, while overexpression of *GmmiR156b* delays flowering under long day and exhibits attenuated expression of *SQUAMOSA PROMOTER BINDING*‐*LIKE (SPL)* genes.

As observed in rice, soybean employs unique mechanisms in its flowering regulation. In addition to *FT’s* well-conserved flowering promoter function, some of its homologs, *GmFT4* (Zhai et al., 2014) and GmFT1a (Liu et al., 2018), are reported to act as inhibitors of flowering. Similarly, the soybean CO homologs *GmCOL1a* and *GmCOL1b*, homologous genes derived from a recent genome duplication event, are observed to delay flowering when they are overexpressed (Cao et al., 2015). Moreover, the major maturity locus *E1* that inhibits flowering and maturity encodes a legume-specific transcription factor carrying a B3 domain (Xia et al., 2012). The *E1* locus is known to possess a predominant effect on flowering and maturity under long day in the genetic background carrying functional *E3* and *E4* alleles. Plants carrying functional *E1* alleles show low expression levels of the flowering promoters *GmFT2a* and *GmFT5a*, as well as high expression of the flowering inhibitor *GmFT4*. Recently, *E1* is shown to be downregulated directly by a homolog of *EARLY FLOWERING 3* (*ELF3*), the causal gene of the maturity locus *J* and a promoter of flowering (Lu et al., 2017). *ELF3* functions in maintenance of circadian rhythms and inhibits flowering in Arabidopsis, whereas in rice, the *ELF3* homolog *Hd17* promotes flowering by attenuating a rice-specific floral inhibitor, *Grain number, plant height and heading date 7* (*Ghd7*) (Zhao et al., 2012).

Despite the above progress, far less is currently known about the flowering genes of soybean than about those of Arabidopsis or rice. In addition, due to functional redundancies and a lack of comprehensive forward-genetic programs, our knowledge of soybean flowering control is skewed toward genes with existing genetic variation that underlie flowering and maturity diversification and regional adaptation. Moreover, characterization of soybean gene functions generally relies on ectopic overexpression, which may lead to changes in a large number of gene cascades. Consequently, the precise modes and molecular mechanisms of regulatory interactions among soybean flowering genes remain largely unknown.

Statistic inference of regulatory interactions based on high-throughput gene expression data has been studied intensely in the bioinformatics and systems biology community. Co-expression and partial correlation analyses (Stuart et al., 2003; de la Fuente et al., 2004) are the most commonly employed approaches to produce an undirected graph in which a pair of genes that show similar expression patterns is connected with an edge. In order to infer gene regulatory networks with directed edges, various inference algorithms, including Boolean, Bayesian, and Factor-Graph network modeling, have been proposed and applied to gene expression data with varied success (Shmulevich et al., 2002; Gat-Viks et al., 2006). Most methods rely on expression data from time series experiments to detect a gene that causally influences a target gene in the presence of a certain time lag. Major issues within this framework include biological and experimental noise, an insufficient sampling rate, the inherent complexity of inferring possible indirect interactions, and changing networks over time and space. As a consequence, these methods have been applied to date only to well-characterized, relatively small networks controlling a narrow range of cell types with extensive prior knowledge. A recently proposed approach that is reported to outperform existing leading methods is the use of sparse linear regression to discover regulator-regulatee gene pairs in a computationally efficient manner in combination with Granger causality heuristics to reduce false positive regulations (Emad and Milenkovic, 2014; Sun et al., 2015). However, several crucial uncertainties remain, including applicability of this approach to a relatively large set of genes in a complex biological event, the influence of noise, and the amount of data and sampling schemes necessary for an accurate estimate of regulatory interactions. Despite such challenges, these algorithmic inference approaches promise a significant possible impact on reverse engineering of previously unknown biological networks.

To better understand photoperiodic flowering regulation in soybean, we elucidated global gene expression patterns under different photoperiod regimes. Transcriptome signatures of the *E* loci showed their unique functions in flowering control and identified candidate genes that may act downstream of the *E* loci. Assisted by our network inference algorithms, we demonstrated the regulatory roles of *GmCOL1a* and *GmCOL1b* in flowering control in the inferred soybean flowering gene network.

## Results

### Transcriptome Signatures of Flowering Regulation in the *E* Loci

**Figure 1:**
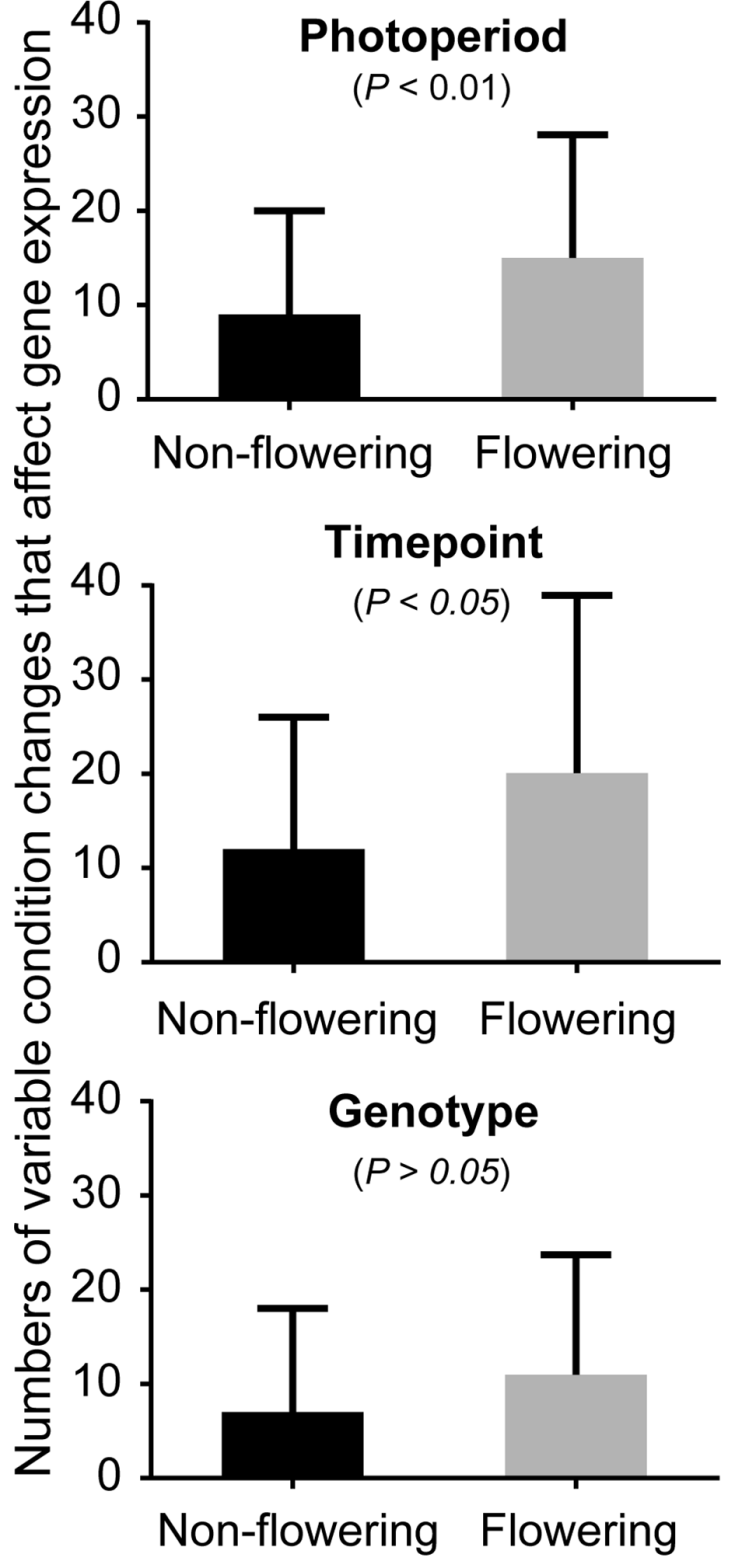
The effects of photoperiod, time point, and genotype on expression of the non-flowering genes and flowering genes. The average numbers of variable conditions in which a gene showed significant differential expression when one variable is fixed (photoperiod, time point, or genotype) for the non-flowering genes (black columns) or the flowering genes (gray columns). For instance, under long-day photoperiod, there are possible variable conditions of 18 (6 genotypes × 3 sampling time points). Since there are three photoperiod regimes, the total number of conditions is 54 under fixed photoperiod. Similarly, the total number of conditions under fixed time point or under fixed genotype is 54. Whitney nonparametric test was employed to examine the statistical significance difference ( *p* > 0.05) in differential expression using *Prism* v.6.0.

Soybean samples were obtained at three time points (T1: 6:30, T3: 14:30, and T5: 22:30) under three photoperiod conditions: short day, long day, and a shift from long day to short day (shift), using Clark, Williams 82, and the near isogenic lines (NILs) of *E1*, *E2*, *E3*, and *E5*. Our transcriptome profiling elucidated the response of soybean genes to photoperiods, sampling time points, and genotypes (**Figure 1**, **Table 1**, and **Supplemental Figures S1**–**S3**). A total of 37,707 genes out of 56,596 genes expressed in our data set responded to at least one of these variables. More than half of these differentially expressed genes responded to all variables (21,257 genes), while 13–14% responded to a single variable (**Table 1**). Sampling time points affected the highest number of genes, followed by photoperiods and genotypes, suggesting that a large number of genes are rhythmically expressed. The soybean homologs of known flowering genes in model plants (flowering genes) and the non-flowering gene homologs (non-flowering genes) showed similar behavior in general (**Supplemental Table S1** and **Table 1**); however, further analysis uncovered higher sensitivity of the flowering genes than non-flowering genes to the given variables (**Figure 1**). By counting how many variable condition contrasts affected expression of a gene when one variable was fixed (photoperiod, time point or genotype), we observed that the flowering genes responded to significantly more variables than the non-flowering genes under fixed photoperiod (*p* < 0.01) and under fixed time point (*p* < 0.05), whereas the difference was marginal under fixed genotype (*p* = 0.06). The observed responses of the flowering genes are consistent with the photoperiodic nature of soybean flowering behavior.

**Table 1:** Numbers of differentially expressed genes in response to different variables.

Variables	Non-flowering genes	Flowering genes
TPG	21,257 (57%)	192 (54%)
TP	9,041(24%)	93 (26%)
TG	1,043 (3%)	8 (2%)
PG	729 (2%)	14 (4%)
T (specific)	2,966 (8%)	27 (8%)
P (specific)	1,250 (3%)	15 (4%)
G (specific)	1,067 (3%)	5 (1%)
T (all)	34,307	320
P (all)	32,277	314
G (all)	24,096	219

Mann-Whitney nonparametric test was employed to examine the statistical significance difference (p > 0.05) in differential expression using Prism v.6.0. T, time point,

P, photoperiod; G, genotype; specific, unique to each variable; all, all genes responding to each variable.

**Figure 2:**
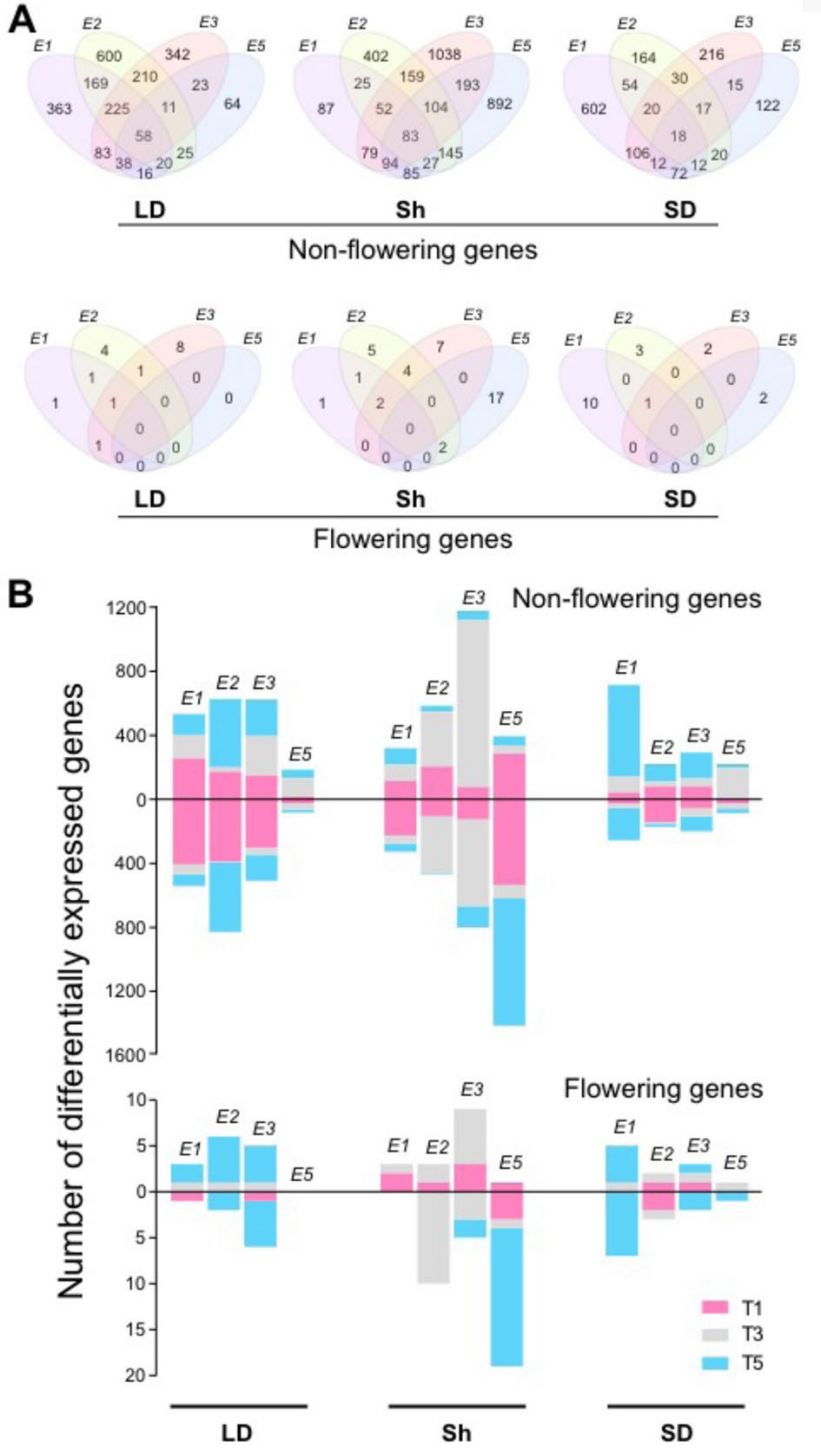
The effects of *E* loci in genome-wide gene expression. **(A)** Venn diagrams indicating the overlaps between *E1*, *E2*, *E3*, and *E5* in differentially expressed non-flowering genes (top) and flowering genes (bottom) under the control of the *E* loci under long day (LD), shift (Sh), and short day (SD). **(B)** Numbers of differentially expressed non-flowering genes (top) and flowering genes (bottom) under the control of *E1*, *E2*, *E3*, and *E5* at the time points T1 (pink), T3 (gray), and T5 (blue) under LD, shift (Sh), and SD. The bars above 0 represent upregulated genes, and the bars below 0 represent downregulated genes.

To understand the mechanisms of *E* loci in photoperiodic flowering regulation, we extracted genes controlled by *E* loci under different photoperiods. The effects of *E* loci were determined from the genotype contrasts as follows: Clark (e*1E2E3e5*) and NIL K65-3366 (*E1E2E3e5*) for *E1*, NIL K65-3366 (*E1E2E3e5*) and NIL L66-432 (*E1e2E3e5*) for *E2*, NIL K65-3366 (*E1E2E3e5*) and NIL L74-441 (*E1E2e3e5*) for *E3*, and Clark (*e1E2E3e5*) and NIL L92-1195 (*e1E2E3E5*) for *E5*. The influence of *E* loci on the transcriptome differed between different photoperiod regimes (**Figures 2A, B**). Under non-flowering inductive long day, *E2* showed the strongest effect on expression of the non-flowering genes, with 1,318 genes expressed differentially, while *E5* showed the least effect, with 255 genes (**Figures 2A, B**). Among the *E2*-regulated genes, 45.5% were uniquely affected by *E2* (**Figure 2A**). A significant portion of the differentially expressed non-flowering genes by *E1*, *E2*, and *E3* appeared at dawn at time point 1, whereas the flowering genes appeared mostly at dusk at time point 5 (**Figure 2B**). Under flowering inductive short day, *E1* exhibited the dominant effect, with 896 affected genes (**Figures 2A, B**). Among these genes, 67.2% were unique to *E1* (**Figure 2A**). The majority of the *E1*-regulated non-flowering genes appeared in the middle of the night at time point 5 (**Figure 2B**). A similar trend in the evening effect of *E1* was seen in the flowering genes. Under the photoperiod shift condition, *E3* and *E5* had the strongest effect with 1,802 genes (57.6% unique to *E3*) and 1,623 genes (55% unique to *E5*), respectively, while *E1* showed the least effect, with 533 affected genes (**Figures 2A, B**). The majority of the *E2*- or *E3*-regulated genes appeared during the day at time point 3 for both non-flowering and flowering genes (**Figure 2B**). Most of the *E5*-regulated genes were downregulated. Uniquely in the flowering genes, a significant portion of the *E5*-regulated genes were differentially expressed at time point 5. Across all photoperiods, time point 1 was underrepresented in the flowering genes compared with that in the non-flowering genes. Although a large number of differentially expressed non-flowering genes were under the control of multiple *E* loci (**Figure 2A**), only a few flowering genes overlapped across *E* loci under long-day and short-day photoperiods, implying discrete flowering gene networks under different *E* loci. Notably, most of the *E5*-regulated non-flowering and flowering genes were differentially expressed under the shift condition (**Figures 2A, B**), suggesting the important role of *E5* in adaptation to changing photoperiods.

Gene ontology (GO) analysis unveiled the unique nature of each *E* locus (**Supplemental Table S2**). Indicating the possible diverse roles of *E1*, the GO terms associated with cell wall biogenesis and phenylpropanoid biosynthesis were enriched in the differentially expressed genes specific to *E1*, while cytokinin-mediated signaling took up 60% of the GO terms associated with the *E2*-specific genes. Many of the E3-specific genes were involved in photosynthesis, and the E5-specific genes were enriched for photoperiodism/flowering.

### Candidate Flowering Genes Controlled by *E* Loci

The flowering genes under the control of *E2* (*GmGIa*) and *E3* (*GmPHYA3*) support the conserved roles of *GI* and *PHYA* in soybean in the control of the circadian clocks and downstream flowering transition (**Supplemental Table S3**). We found homologs of the circadian clock genes *LATE ELONGATED HYPOCOTYL* (*LHY*) and *TIMING OF CAB EXPRESSION 1* (*TOC1*), clock-controlled *CYCLING DOF FACTOR* (*CDF*), the flowering promoters *GmFT2a* and *GmFT5a*, and homologs of the floral meristem identity gene *AGAMOUS-LIKE 8* (*AGL8*/*FUL*) under the control of *E2*, as well as two homologs of *GI*. Under the control of *E3*, we found homologs of the clock genes *LHY* and *PSEUDE RESPONSE REGULATOR* (*PRR*) genes, as well as *CO*, *FT*, *TWIN SISTER OF FT* (*TSF*), and *AGL8*/*FUL*. The causal gene of *E5* is unknown; however, many homologs of circadian clock genes including *LHY* and *PRR*s and clock-influenced flowering genes including *GI*, *CDF*, and *CO* are influenced under the control of *E5* under the shift condition. Most of the *E1*-regulated flowering genes appeared under short day, including homologs of the plant hormone biosynthesis genes *ACC SYNTHASE 10* (*ACS10*) and *GA1*, *FT* homologs, and multiple floral meristem identity genes *AGL8*/*FUL*, *AGL20*, *APETALA 3* (*AP3*), and *PISTILLATA* (*PI*). Unlike *E2*, *E3*, and *E5*, no clock genes appeared differentially expressed under *E1* in our data.

Targeting potential downstream genes of the legume-specific flowering gene *E1*, we conducted quantitative reverse transcription polymerase chain reaction (RT-PCR) analyses using all six time point samples for verification. Several transcription factors displayed clear differential expression in Clark (*e1E2E3e5*) and NIL K65-3366 (*E1E2E3e5*), including the *RELATED TO AP2.7* (*RAP2.7*)/*TARGET OF EARLY ACTIVATION TAGGED 1* (*TOE1*) homolog *GmTOE1b* (Glyma.19G178200), the *AP3* homologs Glyma.01G169600 and Glyma.06G027200, and the *PI* homologs Glyma.04G245500 and Glyma.14G155100 (**Supplemental Figure S4**). *GmTOE1b* (Glyma.19G178200), a homolog of *RELATED TO AP2.7* (*RAP2.7*)/*TARGET OF EARLY ACTIVATION TAGGED 1* (*TOE1*) in Arabidopsis, was expressed higher in Clark carrying recessive *e1* (*e1E2E3e5*) than NIL K65-3366 carrying functional *E1* (*E1E2E3e5*) under both short day and long day, suggesting that the functional *E1* allele suppresses *GmTOE1b*. Overcoming the effect of *E1*, *GmTOE1b* was upregulated by *e2* in NIL L66-432 (*E1e2E3e5*) and by *e3* in NIL L74-441 (*E1E2e3e5*). *GmTOE1b* possesses a miR172 target site conserved among *GmTOE* genes, suggesting that miR172-mediated suppression is a possible mechanism of its downregulation. *GmTOE1b* was expressed ubiquitously across all tissue types tested (**Supplemental Table S5**). These observations suggest that *GmTOE1b* integrates the effects of *E1*, *E2*, and *E3* and that it may act as a flowering promoter. In contrast to *GmTOE1b*, the *AP3* homologs Glyma.01G169600 and Glyma.06G027200 and the *PI* homologs Glyma.04G245500 and Glyma.14G155100 were regulated by *E1* but not by *E2 or E3*. The *AP3* and *PI* homologs were expressed lower in Clark carrying recessive *e1* (*e1E2E3e5*) than in NIL K65-3366 carrying functional *E1* (*E1E2E3e5*) under short day (**Supplemental Figure S4**), suggesting that *E1* may induce the *AP3* and *PI* homologs and that they may act as potential flowering inhibitors. However, while the *AP3* and *PI* homologs were expressed in all tissues except hypocotyl at low levels, their expression in flower buds was strikingly high (**Supplemental Figure S5**), suggesting their potential primary roles in flower development.

### Inference of the Soybean Circadian Clock Network

**Figure 3:**
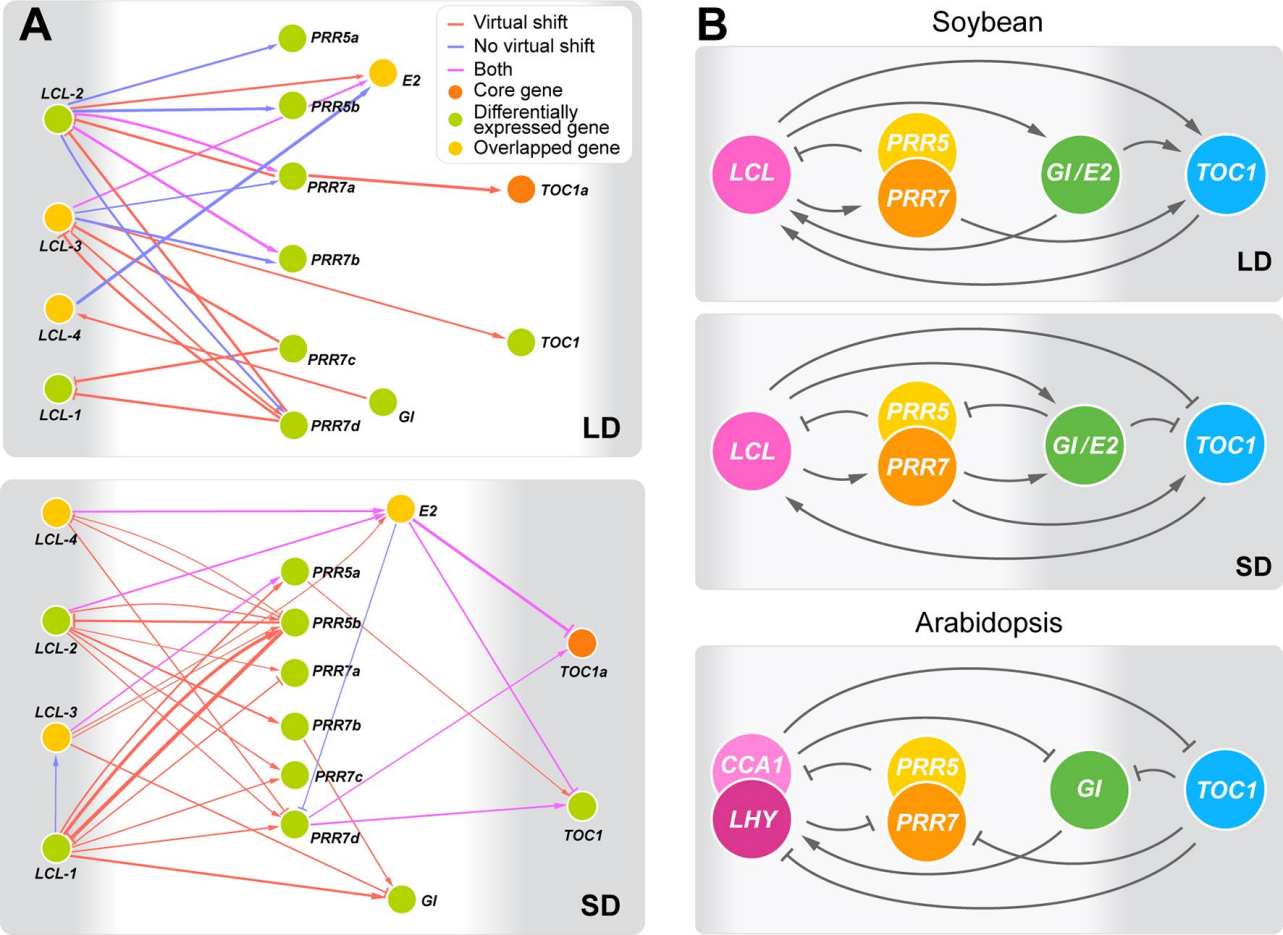
The predicted circadian clock gene networks in soybean. **(A)** Predicted regulatory interactions among the 14 circadian clock gene homologs in the 74 candidate flowering genes (weight ≥ 0.2) under long day (LD) and short day (SD) with or without a virtual time shift that captures regulatory interactions that may occur during the night between time points T5 and T1. The thickness of edges represents confidence levels. Arrowheads and perpendicular lines illustrate induction and repression of transcriptional activity, respectively. **(B)** The outlined predicted circadian clock gene networks in soybean under LD and SD in comparison with the circadian clock gene network in Arabidopsis (Sanchez and Kay, 2016). Arrowheads and perpendicular lines illustrate induction and repression of transcriptional activity, respectively.

Aiming at better understanding of regulatory interactions among flowering genes, we developed CausNet v0.1 (https://github.com/Veggente/soybean-network), a network inference package that combines sparse linear regression and Granger causality algorithms to obtain potential regulatory interactions while mitigating data limitation and false positive interactions. In addition, taking advantage of biological and experimental noise, CausNet conducts perturbation analysis using Gaussian approximation of bootstrapping based on replication data points and provides reliability scores for predicted regulatory interactions, allowing identification of regulatory interactions with high confidence. We ran CausNet with a total of 74 genes, the union of the 61 differentially expressed genes under *E* loci and the 22 core soybean flowering genes obtained from literature whose functions have been experimentally verified in soybean (**Supplemental Table S4**), using the transcriptome data consisting of three time points under long day or short day photoperiod. Among the predicted regulatory interactions, we found a highly intraconnected subnetwork that consisted of the homologs of circadian clock genes connected with moderately high confident edges (0.2 < weight < 0.6) in both long day and short day photoperiods (**Figure 3A**). The subnetwork was distinct from other predicted regulatory interactions. A comparison of this subnetwork with the known circadian clock network in Arabidopsis is shown in **Figures 3B**, (Sanchez and Kay, 2016). Our identification is in twofold. First, the soybean subnetwork bears a general structural resemblance to the Arabidopsis circadian clock network, with multiple feedback regulations between a pair of genes, including *LHY*/*CCA1* (*LCL*) genes and *PRR* genes, *LHY*/*CCA1* (*LCL*) and *E2* (*GmGIa*), and *LHY*/*CCA1* (*LCL*) and *TOC1* (**Figure 3A, B** and **Supplemental Figure S6**). However, it lacked several key genes including *ELF3*, *ELF4*, and *LAX*. Second, despite such similarities, the predicted regulatory roles of some of these genes in the soybean network appear different from that of the known circadian clock genes in *Arabidopsis*. Most of the edges in the Arabidopsis circadian clock network suppress their regulatees, whereas the predicted soybean clock gene network consists of a large number of edges with activation function. Further computational and empirical investigations are necessary, but these observations suggest general structural conservation of the circadian clock gene networks between Arabidopsis and soybean while raising a possibility that species-specific adjustments of the clock network may exist.

### Inference of the Soybean Flowering Gene Network Identified Previously Reported Regulations

**Figure 4:**
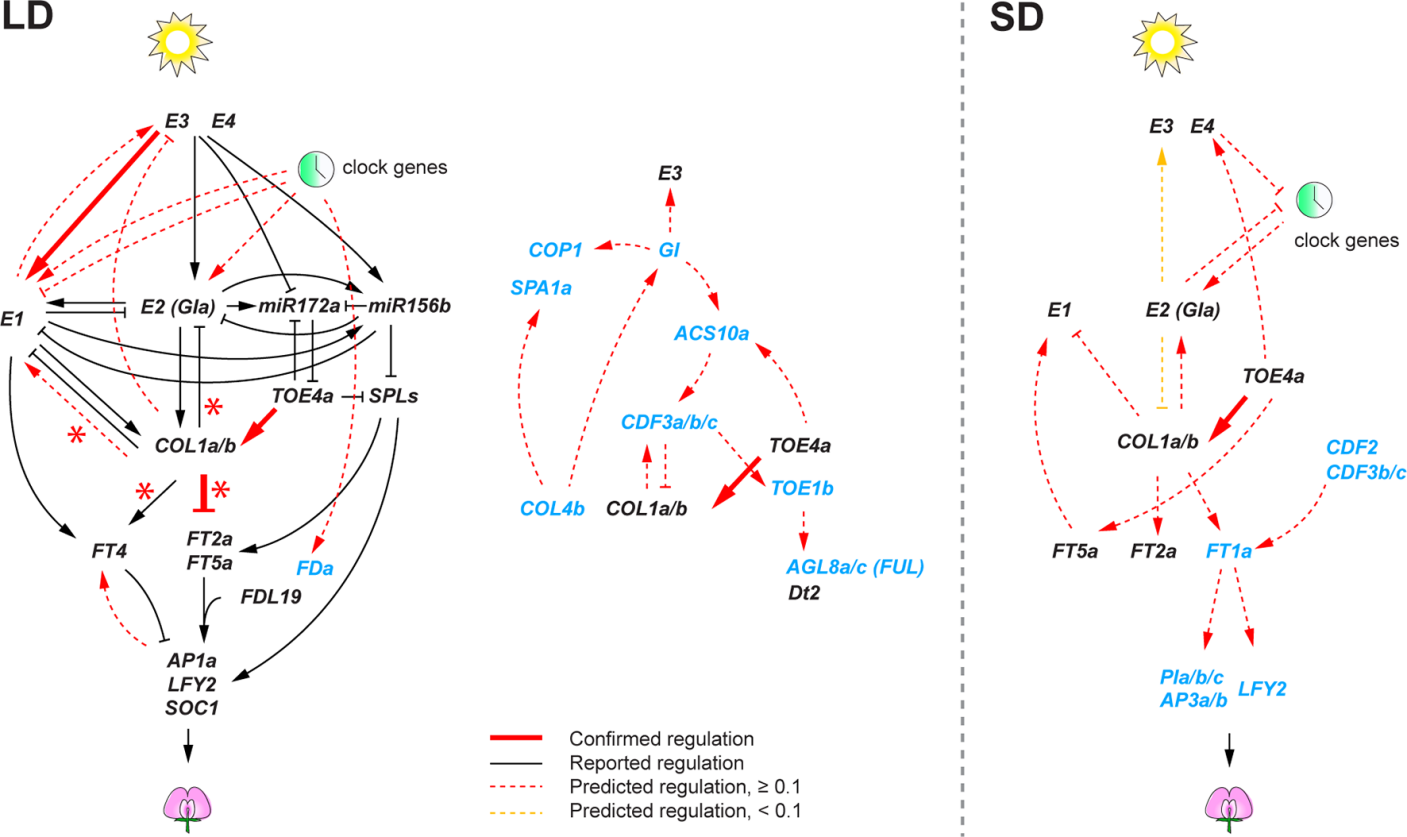
The soybean flowering gene networks consisting of reported regulatory interactions in literature and predicted regulatory interactions under long day (LD) and short day (SD). Predicted regulatory interactions are obtained using the 74 candidate flowering genes with virtual shift. Genes shown in black are previously characterized genes in literature, and genes in blue are differentially expressed genes under *E* loci. Reported interactions are shown as solid edges, and predicted interactions are shown as red dotted or solid edges (weight ≥ 0.1). Red solid edges indicate predicted interactions that are confirmed in reported regulatory interactions in literature. Red asterisks indicate regulatory interactions confirmed in *GmCOL1*- and *GmCOL2*-RNAi transgenic plants. Yellow dotted edges indicate less significant edges (weight < 0.1) in the 74 flowering genes but notably significant (weight ≥ 0.6) when the 22 core flowering genes are used for network prediction. Arrowheads and perpendicular lines illustrate induction and repression of transcriptional activity, respectively.

Contrary to the circadian clock subnetwork, we found a smaller number of high confident regulatory interactions among the remainder of the flowering genes. This likely stems from the design of our transcriptome experiment with samples at three time points in a 24-h period, which would allow identification of regulatory interactions among rhythmically expressed genes, but it is likely less powerful to identify interactions among genes that show weak or no rhythms. Therefore, in the predicted flowering gene networks, we further examined predicted interactions that showed relatively high confidence levels (weight ≥ 0.1) (**Figure 4**). Despite the limitation, we found that some of the predicted regulatory interactions overlapped with the regulations that were previously reported in literature. Most of the literature-confirmed regulatory interactions appeared under flowering non-inductive long day: *E3* activating *E1* (Xu et al., 2015), and *GmCOL1a*/*GmCOL1b* repressing *GmFT2a* and *GmFT5a* (Cao et al., 2015). Notably, however, we found that the inferred role of *GmCOL1a*/*GmCOL1b* in *E1* activation under long day was contradictory to a previous report, in which soybean transgenic plants overexpressing *GmCOL1a* showed low expression of *E1* (Cao et al., 2015). The inferred interaction in which *GmTOE4a* activating *GmCOL1a*/*GmCOL1b* was confirmed under both long day and short day (Zhao et al., 2015). The lack of confirmed interactions under short day is largely due to the lack of reported interactions under short day in literature. Consistently with the photoperiodic nature of soybean flowering control, the inferred flowering gene networks showed a sharp contrast between long day and short day. Although several conserved key genes appeared in both photoperiods, most of their predicted interactions under long day did not appear under short day or vice versa; in some cases, they showed opposite function between long day and short day. For example, *E1* activation by *E2*, *E3*, and clock genes in the long-day network was missing in the short-day network. *GmCOL1a*/*GmCOL1b* genes also showed opposite functions; under long day, *GmCOL1a*/*GmCOL1b* activated *E1* and suppressed *FT2a* and *FT5a*, while under short day, *GmCOL1a*/*GmCOL1b* suppressed *E1* and activated the *FT* genes.

### Empirical Confirmation of the Inferred Regulatory Roles of *GmCOL1a* and *GmCOL1b*

**Figure 5:**
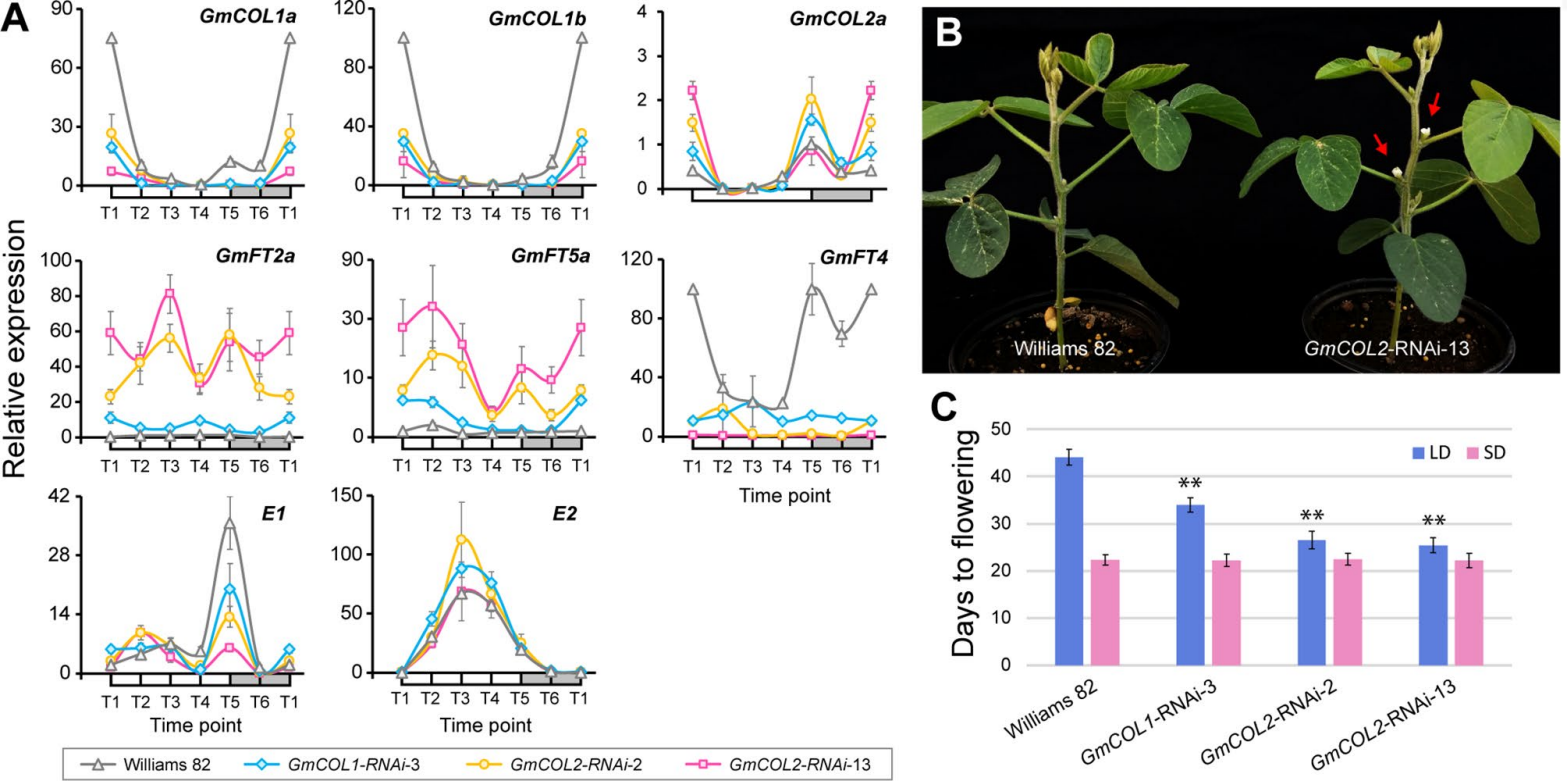
*GmCOL1*-RNAi and *GmCOL2*-RNAi transgenic soybean plants. **(A)** Real-time quantitative reverse transcription polymerase chain reaction (RT-PCR) analysis of *GmCOL1a*, *GmCOL1b*, *GmCOL2a*, *GmFT2a*, *GmFT5a*, *GmFT4*, *E1*, and *E2* expression levels in transgenic lines *GmCOL1*-RNAi-3, *GmCOL2*-RNAi-2, and *GmCOL2*-RNAi-13 in a 24-h time course under long day (LD) (16 h light) at 22 days after germination. Three biological replications were sampled from the fully expanded trifoliates for each time point. Error bars indicate standard deviation. **(B)** Representative early flowering phenotype of the *GmCOL*-RNAi plants (shown is *GmCOL2*-RNAi-13) at 25 days after emergence under LD (16 h light). Red arrows indicate flower buds. **(C)** Flowering time of three independent transgenic lines: *GmCOL1*-RNAi-3, *GmCOL2*-RNAi-2, and *GmCOL2*-RNAi-13 under LD (16 h light) and short day (SD, 10 h light). Flowering time was assessed as the number of days from germination until emergence of the first flower. At least 10 plants were measured for each line (n ≥ 10). Error bars indicate standard deviation. **Significant difference (*p* ≤ 0.01) compared with control (Williams 82).

Because of the striking contrast, we focused on *GmCOL1a* and *GmCOL1b* and created transgenic soybean plants exhibiting reduced expression of these genes *via* RNA interference (RNAi) in order to experimentally verify their predicted functions (**Figures 5A–C**). Among several independent transgenic lines, a single *GmCOL1*-RNAi line exhibited strong reduction of *GmCOL1a*/*GmCOL1b* expression (**Figure 5A**). Under non-inductive long day, the flowering promoters *GmFT2a* and *GmFT5a* were upregulated in *GmCOL1*-RNAi plants, providing empirical support for the predicted role of *GmCOL1a*/*GmCOL1b* in suppression of *GmFT2a* and *GmFT5a*. In addition, *GmCOL1*-RNAi plants showed attenuated expression of the flowering repressor *GmFT4*, supporting that *GmCOL1a*/*GmCOL1b* activate *GmFT4*. These verified regulatory interactions are consistent with literature (Cao et al., 2015). *GmCOL1*-RNAi plants flowered significantly earlier than wild-type plants (Williams 82) by 10 days under long day (*p* < 0.01) (**Figures 5B, C**), whereas no clear flowering time effect was observed under short day (**Figure 5C**). These observations confirm *GmCOL1a* and *GmCOL1b* function as a flowering inhibitor through suppression of *GmFT2a* and *GmFT5a* and activation of *GmFT4* under long day. Moreover, *GmCOL1*-RNAi clarified the inferred roles of *GmCOL1a*/*GmCOL1b* in the regulation of *E1*. In the inferred flowering gene network under long day, *GmCOL1a*/*GmCOL1b* activate *E1* expression (**Figure 4**), contradicting to the reported suppression of *E1*. We found that peak expression of *E1* was dampened in *GmCOL1*-RNAi plants (**Figure 5C**), supporting the predicted regulation. In addition, we found that *GmCOL1*-RNAi plants showed higher peak expression of *E2*, confirming the previously reported role of *GmCOL1a*/*GmCOL1b* in downregulation of *E2* (Cao et al., 2015).

The roles *GmCOL1a*/*GmCOL1b* in *E1* activation and *E2* downregulation were also supported by cross-interference of *GmCOL1a*/*GmCOL1b* in *GmCOL2*-RNAi plants (**Figures 5A–C**). In the two independent *GmCOL2*-RNAi lines, we observed strong reduction of *GmCOL1a*/*GmCOL1b* expression but no reduction of *GmCOL2a*/*GmCOL2b*, suggesting that *GmCOL2-*RNAi cross-targeted *GmCOL1a* and *GmCOL1b*. This cross-interference occurred likely due to hundred- to thousand-fold higher expression levels of *GmCOL1a*/*GmCOL1b* than *GmCOL2a*/*GmCOL2b* expression, leading to efficient amplification of interference effects for *GmCOL1a*/*GmCOL1b*.

### The Feed-Forward Loop Involving *GmCOL1a/GmCOL1b* and *E1* Causes Enhanced Late Flowering

**Figure 6:**
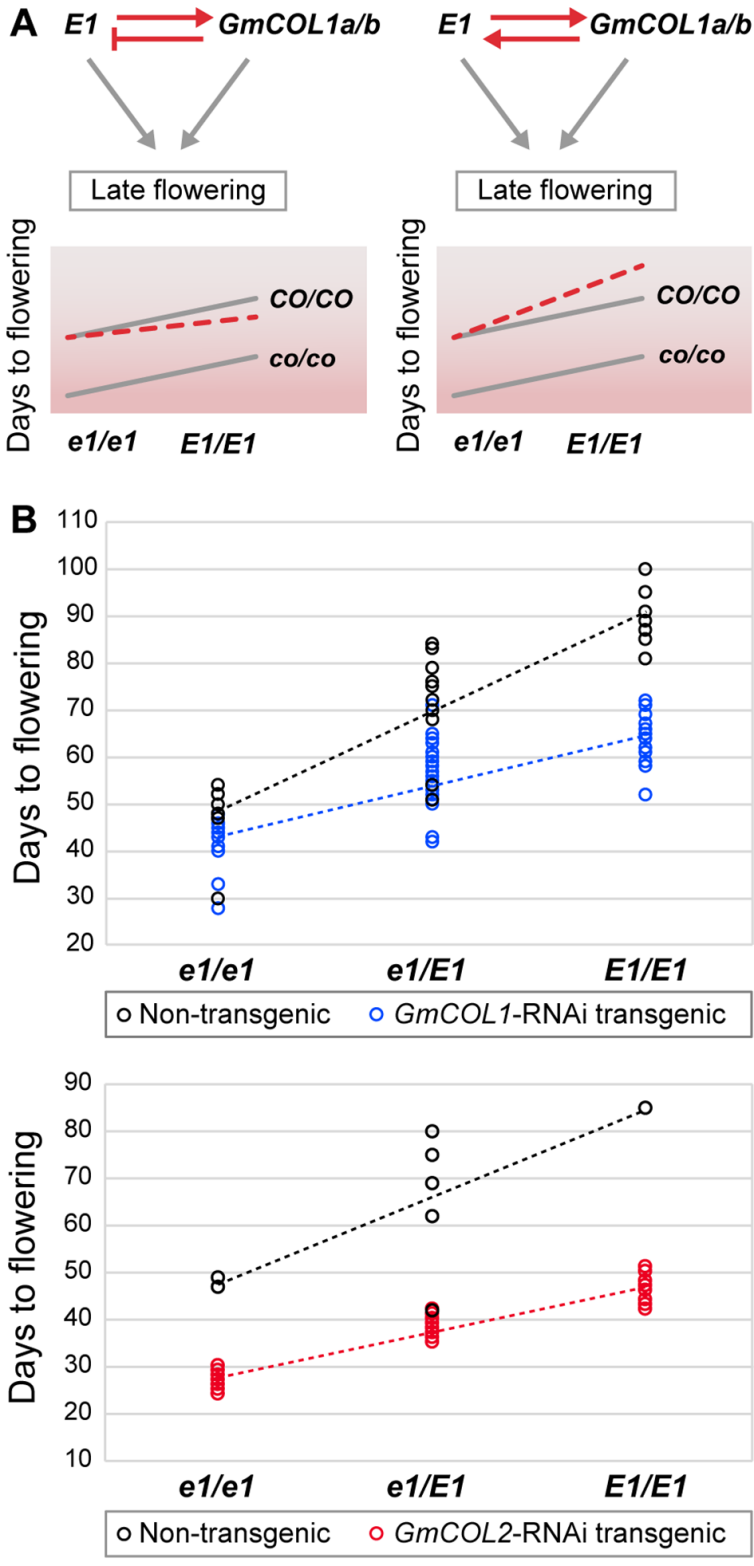
The function of the feedback regulation between E1 and *GmCOL1a/GmCOL1b* in flowering time control. **(A)** In a feedback regulatory interaction in which E1 upregulates *GmCOL1a/GmCOL1b* and *GmCOL1a/GmCOL1b* downregulates *E1* (left), both *E1* and *GmCOL1a*/*GmCOL1b* will be diminished when both genes are functional, leading to earlier flowering than the additive value of functional *E1* and functional *GmCOL1a/GmCOL1b*. In a feedback regulatory interaction in which *E1* and *GmCOL1a/GmCOL1b* upregulate each other (right), *E1* and *GmCOL1a/GmCOL1b* upregulation will be accelerated when both genes are functional, leading to later flowering than the additive value of *E1* and *GmCOL1a/GmCOL1b*. The parallel gray lines represent a pattern of additive interaction between *E1* and *GmCOL1a/GmCOL1b*. **(B)** Genetic interaction of *E1* and *GmCOL1a/GmCOL1b* in flowering time control. Flowering time was measured in the segregating F2 population obtained from a cross between the *E1* NIL and *GmCOL1*-RNAi (top) or *GmCOL2*-RNAi (bottom) under long day (16 h). Flowering time was assessed as the number of days from germination until emergence of the first flower. Each circle represents an individual plant in the F2 populations.

The contradiction between the inferred promoter function of *GmCOL1a*/*GmCOL1b* in *E1* expression and their reported inhibitor function under long day led us to genetically examine the interaction between *GmCOL1a*/*GmCOL1b* and *E1*. Feedback regulations between *GmCOL1a*/*GmCOL1b* and *E1* create a composite of two feed-forward loops that would affect their functional interaction in their activation of the flowering inhibitor *GmFT4* to cause delayed flowering (**Figure 6A**). If *GmCOL1a*/*GmCOL1b* acts as an inhibitor of *E1* expression as reported in literature, *E1* will be dampened by *GmCOL1a*/*GmCOL1b* over time; hence, we expect that the late flowering effect of *GmCOL1a*/*GmCOL1b* and *E1* will be less than their additive value. In contrast, if *GmCOL1a*/*GmCOL1b* acts as a promoter of *E1* as identified in this work, we expect that the late flowering effect of *GmCOL1a*/*GmCOL1b* and *E1* will be enhanced in a synergistic manner. To test these possibilities, we made a cross between *GmCOL1*-RNAi plants carrying recessive *e1* and the NIL K65-3366 carrying functional *E1* and examined flowering time phenotypes of the plants in the segregating F2 population under long day (**Figure 6B**). In the absence of functional *E1* (*e1*/*e1*), *GmCOL1*-RNAi plants flowered approximately 6 days earlier than non-RNAi plants, while in the *GmCOL1*-RNAi background, the *E1* NIL (E1/E1) plants showed later flowering than the *e1*/*e1* plants, confirming that *GmCOL1a*/*GmCOL1b* and *E1* can function partly independently through separate genetic pathways. We observed synergistic interaction of *GmCOL1a*/*GmCOL1b* and *E1* in their late flowering actions. The plants carrying both functional *GmCOL1a*/*GmCOL1b* and the *E1* NIL showed enhanced late flowering in a synergistic fashion rather than additive, supporting the positive feedback regulations between *GmCOL1a*/*GmCOL1b* and *E1* that ensure the strong delay of flowering transition in the long-day flowering network. Confirming this observation, a similar synergistic late flowering trend appeared in the F2 population obtained from a cross between *GmCOL2*-RNAi plants and the *E1* NIL. Thus, our network inference successfully identified the positive regulatory interaction of *GmCOL1a*/*GmCOL1b* with *E1*, and we conclude its biological role in flowering suppression.

## Discussion

### Transcriptome Signatures of the Mechanisms of Photoperiodic Control in Soybean

Our transcriptome profiles of *E* loci NILs revealed the importance of a specific phase of the day, time point 5: dusk under long day and early evening under short day (**Figure 2B**). A large portion of flowering genes were affected by *E2* and *E3* at dusk under long day, while the effects of *E1* on flowering genes were most prominent in early evening under short day. In addition, the majority of *E5*-regulated flowering genes appeared at this time point under the shift condition. These observations indicate that *E* loci activities and varying photoperiods converge during this phase in flowering control. The importance of this phase in flowering control is consistent with a previous report (Xu et al., 2015) that light exposure during this phase is key to induction of *E1* expression and inhibition of flowering. Together, these observations point to the presence of the external coincidence mechanism where internal rhythms and external seasonally changing photoperiods integrate during this light-sensitive phase. In Arabidopsis, CO is known to play central roles in this mechanism (Song et al., 2015). *CO* is rhythmically expressed under both long day and short day and integrates multiple factors including GI and FKF1 that upregulate *CO* mRNA accumulation and CRY2 and PHYA that stabilize CO protein in late afternoon under flowering inductive long day, leading to upregulation of *FT* expression toward dusk. Similarly in soybean, we demonstrate that GmCOL1a/GmCOL1b are necessary for upregulation of *FT*, with the flowering inhibitor *GmFT4* under non-inductive long day, using the network inference algorithms and *GmCOL1*-RNAi plants (**Figures 4** and **5**). In addition, we show that *GmCOL1a*/*GmCOL1b* also upregulate another major flowering inhibitor *E1* during this phase under long day. These observations suggest that *GmCOL1a*/*GmCOL1b* may well be a point of external coincidence in soybean. High accumulation of *GmCOL1a*/*GmCOL1b* transcripts occurs only after dusk, during the night through the morning under both long day and short day (Wu et al., 2014). A plausible hypothesis is that mechanisms similar to Arabidopsis may operate in soybean, in which GmCOL1a/GmCOL1b proteins accumulate and stabilize during the day and induce the flowering inhibitors during the light-sensitive phase at dusk under long day, assisted by other factors that are activated toward dusk, whereas under short day, the absence of GmCOL1a/GmCOL1b proteins or the lack of other cooperating factors may lead to attenuation of the flowering inhibitors during the early evening phase, resulting in flowering induction.

### Transcriptome Signatures of the Roles of the *E* Loci

Despite *E1*’s faint expression and weak flowering effects under short day, many flowering genes are under the control of *E1* under short day (**Figure 2** and **Supplemental Table S3**), indicating the roles of *E1* under flowering inductive photoperiod. Interestingly, among these *E1*-regulated flowering genes, floral meristem identity genes including *AGL*, *AP3*, and *PI* homologs are more enriched than among those under the control of other *E* loci, suggesting that a point of *E1*’s action in the flowering gene network may be closer to these downstream genes than the acting points of *E2*, *E3*, and *E5*. Transcriptomic patterns highlight notably distinct behaviors of *E1* and *E5*. Transcriptomic effects of *E5* suggest its potential roles in response to photoperiod shift (**Figure 2**). The *E5* locus was originally reported more than a quarter century ago (McBlain and Bernard, 1987) and shown to locate nearby *E2* (Watanabe et al., 2011), but its genetic nature has been mysterious. The causal gene of *E5* has not been identified, and its genetic effect is reported to be variable depending on mapping populations, in some cases undetectable (Dissanayaka et al., 2016), raising a question that *E5* may be an allele of the *E2* locus. However, the molecular phenotype of *E5* at the transcriptome level demonstrates that the *E5* NIL possesses a unique locus other than *E2*, *E1*, or *E3*. It is possible that *E5* may be influencing plant adaptation to seasonal changes of photoperiods, an important new trait for future exploitation in breeding programs.

### The Roles of the Composite Coherent Feed-Forward Loop Involving *Gmcol1a/Gmcol1b* and *E1*

We discovered that the flowering inhibitors *GmCOL1a*/*GmCOL1b*, *E1*, and *GmFT4* together comprised the composite coherent feed-forward loop and demonstrated its biological function in the suppression of flowering transition. A feed-forward loop is known to cause a delay in its output (Mangan and Alon, 2003). In the soybean flowering gene network, the *GmCOL1a*/*GmCOL1b*, *E1*, and *GmFT4* feed-forward loop may thus allow increased suppression of flowering transition lasting over the summer. In addition, the positive feedback loop between *GmCOL1a*/*GmCOL1b* and *E1* activating each other would enhance the output flowering suppression, as demonstrated in the synergistic late flowering effect of the functional *GmCOL1a*/*GmCOL1b* and *E1* alleles in combination (**Figure 6**). This finding underscores the importance of the strategy recurrently employed by short-day flowering species: the suppression of flowering transition by multiple flowering inhibitors under non-inductive long day. The inhibition strategy may have been advantageous for short-day plants that set the seeds in fall over a simpler activation strategy in which flowering inducers are upregulated by directly responding to seasonal environmental cues, likely because the former strategy would allow better control and robustness over environmental fluctuations, averting leaky early flowering to ensure sufficient vegetative growth and favorable conditions for seed maturity in fall.

### Transcriptome-Enabled Network Inference

The complexity of the flowering gene network is multifold. The flowering gene network consists of diverse classes of proteins and biochemical regulations in addition to transcriptional regulations, including phosphorylation and protein degradation mechanisms, as well as non-coding small RNAs. It also involves systemic transmission of signals orchestrated at the whole plant level over multiple seasons or years, triggered by multiple environmental and internal cues. Despite such complexity and heterogeneity of the flowering gene network, our network inference approach using transcriptome data solely successfully identified several true regulatory interactions that were experimentally verified. This approach is especially powerful for functional studies of non-model organisms for better understanding of complex biological processes. Our work also points out the importance of experimental design for transcriptome-based network inference, in particular, selection of most appropriate sampling time course suitable for target biological events. Given the fact that the flowering gene network functions in a diurnal as well as developmental time scale, the use of multidimensional time axes may significantly improve inference of the flowering gene network, while the clock gene network may benefit from more dense diurnal time points alone. Despite the apparent needs for better strategies to infer temporal and spatial regulatory interactions and for further empirical verifications, this study provides a foundation for *de novo* reverse engineering of complex biological networks controlling important agronomic traits in crops and informs next challenges for future plant improvement.

## Materials and Methods

### Plant Growth Condition and Sampling

Seeds of soybean genotypes were provided by the USDA Soybean Germplasm Collection (**Table 2**). Two common North American cultivars, Clark (PI 548533) and Williams 82 (PI 518671), and the four NILs of *E* loci (PI 547431, PI 547432, PI 547610, and PI 591490) carrying contrasting alleles in *E1*, *E2*, *E3*, and *E5* in the Clark genomic background were used. The alleles of *E1*, *E2*, *E3*, and *E4* were verified by PCR or DNA sequencing with specific primers (**Supplemental Table S5**) (Xu et al., 2013; Langewisch et al., 2014). The recessive alleles of *E1*, *E2*, and *E3* were identified as *e1-as*, *e2*, and *e3-tr*, respectively.

**Table 2:** Soybean inbred lines and near isogenic lines (NILs) of the maturity loci *E1*, *E2*, *E3*, and *E5* used in this study.

Variety	*E* loci alleles	Days to flowering		
		Field	Greenhouse	
			SD	LD
Clark (PI 548533)	e1E2E3E4e5	33	27 ± 1	63 ± 2
Williams 82 (PI 518671)	e1E2E3E4	33	27 ± 1	61 ± 3
K65-3366 (PI 547431)	E1e2E3E4e5	61	29 ± 2	97 ± 10
L66-432 (PI 547432)	E1e2E3E4e5	50	29 ± 1	74 ± 4
L74-441 (PI 547610)	E1e2e3E4e5	56	30 ± 1	52 ± 3
L92-1195 (PI 591490)	e1E2E3E4E5	36	28 ± 0	70 ± 9
				

The NILs are in the Clark background. The flowering time data in the field is the average of two consecutive years based on the record in the USDA soybean germplasm collection. The flowering time data in the greenhouse is obtained from 20–25 plants per genotype under short day (SD) or long day (LD).

Plants were grown in the greenhouse under short day (10 h of light, 6:45–16:45) and long day (16 h of light, 6:45–22:45) conditions at 25°C and were sampled every 4 h at six time points, T1–T6 (6:30, 10:30, 14:30, 18:30, 22:30, and 2:30), over a 24-h time period 3 weeks after germination. For a shift experiment, plants were first grown under long day for 3 weeks and then transferred to short day for 5 days. A whole shoot above the cotyledon, including three to four trifoliates, stem, and shoot meristems, was harvested from each plant. Three to four biological replications were sampled for each time point and photoperiod condition.

### Transcriptome Sequencing and Analyses

In this study, we analyzed the sequencing data of 162 soybean samples that consisted of the six genotypes, three time points (T1: 6:30, T3: 14:30, and T5: 22:30), and three photoperiod treatments: short day, long day, and a shift from long day to short day (shift) with three biological replications. RNA preparation, Illumina sequencing, and quality check were conducted as described previously (Wu et al., 2014).

The following procedures were run on the *Biocluster* in the Institute for Genomic Biology at the University of Illinois at Urbana-Champaign. We implemented the protocol with *TopHat* and *Cufflinks* (Trapnell et al., 2012) for the differential gene expression analysis of our soybean RNA-seq data. We used the soybean gene models (*Glycine max Wm82.a2.v1*) retrieved from *www.phytozome.net* as the reference. First, we ran *TopHat* to align the sequencing reads to the reference genome with the annotation GTF file. Next, we ran *Cufflinks* to assemble transcripts for each sample. After obtaining the assembled transcripts from *Cufflinks*, *Cuffcompare* was used to compare them to the reference annotation. Finally, we ran *Cuffdiff* with the output files of *Cuffcompare* in order to find significant changes in transcript expression among the examples of different condition.

The potential soybean orthologs of 215 Arabidopsis flowering genes (**Supplemental Table S1**) were obtained by performing a reciprocal *BLASTP* search using soybean gene models from *www.phytozome.net* (*Glycine max Wm82.a2.v1*) that contains 88,647 transcripts with alternative splicing variants, and Arabidopsis gene models from the Arabidopsis Information Resource (*www.arabidopsis.org*). The repeated or low complexity regions from the soybean and Arabidopsis sequences were removed by generating the masking information with *segmasker* and created BLAST databases with the masking information using the FASTA sequence files of soybean and Arabidopsis using *makeblastdb*. *BLASTP* search was carried out using soybean sequences as query and Arabidopsis sequences as target, and *vice versa*. The reciprocal best hits (RBHs) were filtered by cut-off values of *e* value ≤1*e*−5, sequence identity ≥40%, and query or subject coverage ≥50%, resulting in 417 soybean homologs of Arabidopsis flowering genes. Including soybean unique flowering genes *E1* and its homologs, we obtained the 420 soybean flowering genes (**Supplemental Table S1**). The identified flowering genes and the non-flowering genes were used for further analyses comparatively. The response of flowering and non-flowering genes to the variables photoperiods, time points, and genotypes was obtained by Mann-Whitney nonparametric test using *Prism* v.6.0. Functional information of the gene sets that were differentially expressed in response to the allele types of *E1*, *E2*, *E3*, or *E5* was obtained using GO annotations using the GO annotation tool on SoyBase (*www.soybase.org*). GO terms that were significantly more frequent in the gene sets under the control of *E* loci than in the total soybean genes with 95% confidence (*p* < 0.05) were considered enriched.

### Quantitative RT-PCR

For the tissue-specific expression, Williams 82 (PI 518671) was grown in the greenhouse under short day conditions (10 h of light, 6:45–16:45) at 28°C for 30 days before being sampled at 13:00 with three biological replications. For gene expression in *GmCOL1*- and *GmCOL2*-RNAi transgenic soybeans, plants were grown under long day (16 h of light, 6:45–22:45) conditions at 28°C and were sampled every 4 h at six time points, T1–T6 (6:30, 10:30, 14:30, 18:30, 22:30, and 2:30), over a 24-h time period 22 days after germination. Three biological replications were sampled from the fully expanded leaves for each time point. RNA preparation and cDNA preparation were conducted as described previously (Wu et al., 2014). The quality of the cDNA was tested by RT-PCR using a pair of primers (**Supplemental Table S5**) specific for the reference gene *GmPBB2* (Glyma.14G014800).

Quantitative RT-PCR (qPCR) was performed using a StepOnePlus^™^ Real-Time PCR System (Applied Biosystems, Carlsbad, CA, USA), following the manufacture’s manual. All reactions were carried out in a MicroAmp^®^ Fast Optical 96-Wells reaction plate (Applied Biosystems, Carlsbad, CA, USA) plus optical adhesive film (Applied Biosystems, Carlsbad, CA, USA) with a volume of 15 μl per well which consisted of 7.5 μl 2 × Power SYBR Green Master Mix (Applied Biosystems, Carlsbad, CA, USA), 6.4 μl sterilized distilled water, 0.3 μl of 10 M each primer, and 5 μl diluted template. The resulting data were recorded and analyzed by the StepOne^™^/StepOnePlus^™^Software v2.3 (Applied Biosystems, Carlsbad, CA, USA). The following genes were examined with specific primers (**Supplemental Table S5**). Transcript levels were calculated relative to that of the reference gene *ACTIN11* (Glyma.02G091900) with specific primers (**Supplemental Table S5**).

### Soybean Transformation, Crossing, and Flowering Time Measurement

*GmCOL1b*- or *GmCOL2b*-specific 300–400 bp fragments were amplified from the C-terminus of *GmCOL1b* and *GmCOL2b* cDNAs (**Supplemental Figure S7**) and cloned into the pB7GWIWG2(II),0 vector. These constructs were used for transformation of Williams 82 (PI 518671) by the Plant Transformation Facility at Iowa State University. For screening transformants, T1 plants were grown in the greenhouse and were sprayed with herbicide glufosinate (Liberty; Bayer Crop Sciences, Research Triangle, NC, USA) at least three times. The presence of the transgenes in T1 and T2 plants was confirmed by PCR using vector-specific and gene-specific primers (**Supplemental Table S5**).

For crossing, Clark *E1* NIL K65-3366 (PI 547431) was used as the maternal parent, and the homologous transgenic line (*GmCOL1*-RNAi-3 or *GmCOL2*-RNAi-2) was used as the paternal parent. In the segregating F2 generation, the presence of the transgenes was genotyped by PCR using specific primers (**Supplemental Table S5**) and the allele types of *E1* as described previously (Tsubokura et al., 2014). Flowering time measurement was conducted in the greenhouse under long day (16-h light) and short day (8-, 10-, and 12-h light) at 28°C. The date of the first flower seen after emergence is regarded as the day of flowering.

### CausNet Algorithms and Network Inference

The network inference algorithm package CausNet v0.1 creates a directed graph with vertices being genes and weighted edges being regulations, where each edge is associated with two weights indicating confidence levels in the existence and in the function of the edge (activation or repression). CausNet is based on a discrete-time model where the production rate of the mRNA of a gene is the sum of a linear combination of the expression levels of its regulators by fixed coefficients and a Gaussian random nominal production rate. The regulatory interactions are assumed to be sparse in the sense that the number of regulators of a target gene is bounded by a small integer. The main algorithms are adopted from Emad and Milenkovic (2014) and Sun et al. (2015) that consist of two stages: sparse linear regression (Stage I) to discover potential regulator genes for each regulatee gene, and a Granger causality test (Stage II) to remove the false positive regulations. The third stage is a perturbation analysis to estimate the accuracy of the estimated regulations, where random perturbations are generated from the original replication data points and the main algorithms are run on each set of perturbation data independently. Finally, the results from each run are aggregated into a single weighted network. The obtained network is visualized using Cytoscape (Shannon et al., 2003). CausNet v0.1 is deposited to GitHub (https://github.com/Veggente/soybean-network) and freely available. CausNet performance was validated using the Arabidopsis flowering network (Supplemental Text). The input data used in this study are the normalized transcriptome levels of the selected 74 soybean flowering genes under the 18 photoperiod-genotype combinations at three sampling time points in a day (T1: 6:30, T3: 14:30, and T5: 22:30) with three biological replications. The details of the three-stage analyses are explained below.

Stage I: Sparse linear regression. For each regulatee gene, its expression profile is approximated by the best linear combination of the expression profiles of k+1 genes (the target gene and k other genes) with shifted time. In other words, in this stage the algorithm finds k genes for each target gene such that the k+1 genes at time point T1 and time point T3 predict the target gene at time points T3 and T5, respectively, with the minimum residual sum of squares. The type of regulation is determined by the sign of the regression coefficient: a positive coefficient indicates activation, and a negative one repression. Note that in order to capture regulatory interactions that occur during the night, time point T1 was used as a virtual time shift for time point T5. The linear regression is sparse because k is usually much smaller than the total number of genes (k = 3 is used in this study).

Stage II: Granger causality test. For each regulatee gene, the k potential regulator genes are tested one by one as follows. An F statistic is calculated based on the regression errors with and without the chosen potential regulator gene (i.e., k genes vs. k+1 genes, both including the regulatee gene). The potential regulator gene is then rejected if the *p* value associated with the F statistic is below the preset significance level (0.05 is used in this study). A rejection indicates this chosen gene does not have significant contribution towards the regulatee gene in addition to the other k-1 potential regulator genes.

Stage III: Perturbation analysis. We generate truncated Gaussian random expression level data according to the mean and sample variance of the original three replication data and run the above algorithms on each set of independently generated data (a perturbation). This procedure is repeated 100 times. The resulting 100 reconstructed networks are then combined into a single network where the weights for the confidence in the existence and in the type of the regulations are given by the number of occurrences among all the perturbations. Note that the perturbation analysis is a Gaussian approximation of bootstrapping, a classic resampling method used to measure accuracy. It coincides with bootstrapping with resampling size 2 in the sense that both techniques generate random data, and the means and the variances are the same, while the perturbation analysis has a larger support of the data distribution.

## Data Availability Statement

The transcriptome sequencing data were deposited in the Gene Expression Omnibus (GEO) site at the National Center for Biotechnology Information (NCBI) website (the accession numbers GSM1234545 - GSM1234733; www.ncbi.nlm.nih.gov/geo/).

## Author Contributions

YH conceived the project ideas and designed the experiments. WP performed the plant sampling and transcriptome library preparation. MW and WH performed the transcriptome analysis. FW performed the gene expression and transgenic experiments. XK and BH developed the network inference algorithm package. XK performed the network inference analyses under the supervision of BH. FW interpreted the inferred networks. YH wrote the manuscript with input from MW, FW, XK, and BH.

## Funding

This work was supported by the Agriculture and Food Research Initiative Competitive Grants Program from the USDA National Institute of Food and Agriculture (USDA-NIFA2011-00078) to YH and the Plant Genome Research Program from the National Science Foundation (NSF- IOS-PGRP-1823145) to BH and YH.

## Conflict of Interest

The authors declare that the research was conducted in the absence of any commercial or financial relationships that could be construed as a potential conflict of interest.
