# Supplementary material for: Transcriptome-Enabled Network Inference Revealed the GmCOL1 Feed-Forward Loop and Its Roles in Photoperiodic Flowering of Soybean
Source: Front Plant Sci. 2019 Nov 8;10:1221. doi: 10.3389/fpls.2019.01221 (PMC6856076; doi:10.3389/fpls.2019.01221)
Supplement: Supplementary file 2 [file DataSheet_1.pdf]

Supplemental Figure S1

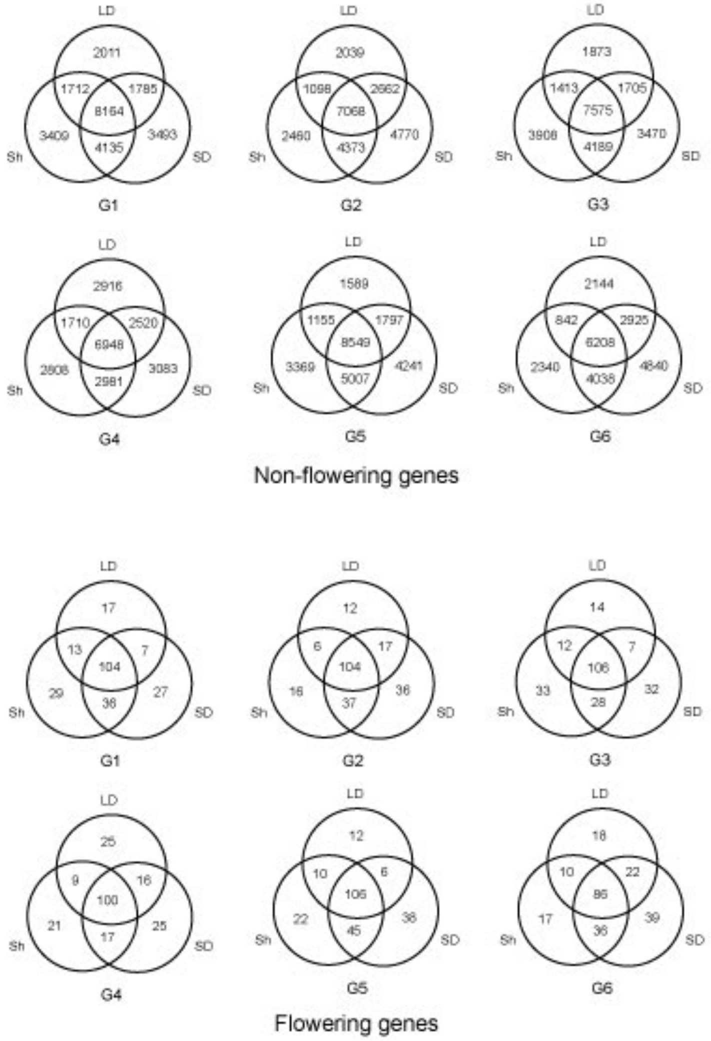

G1	Clark	*e1E2E3E4e5*
G2	Williams 82	*e1E2E3E4*
G3	K65-3366	*E1E2E3E4e5*
G4	L66-432	*E1e2E3E4e5*
G5	L74-441	*E1E2e3E4e5*
G6	L92-1195	*e1E2E3E4E5*

Supplemental Figure S1. Venn diagrams of differentially expressed non-flowering genes (top) and flowering genes (bottom) indicating the overlaps under long day (LD), shift (Sh) and short day (SD) in different genotypes.

Supplemental Figure S2

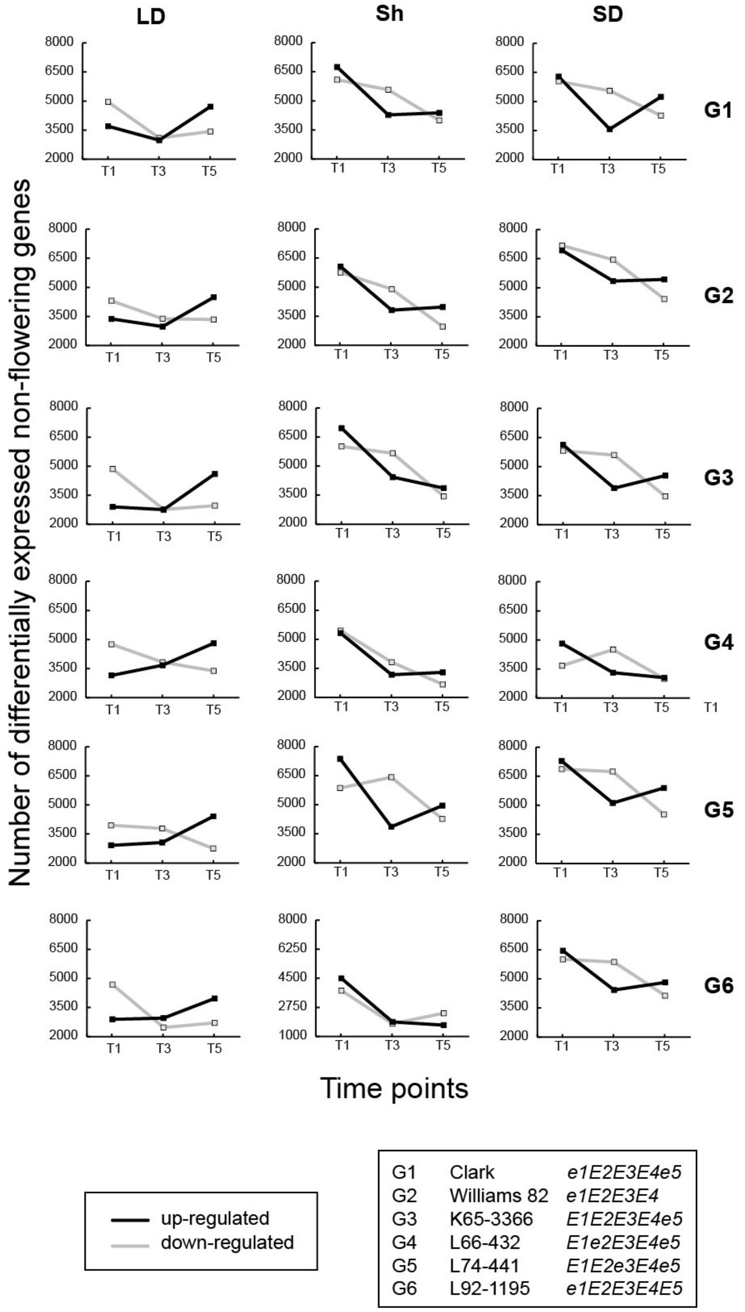

Supplemental Figure S2. Numbers of differentially expressed non-flowering genes at time points T1, T3 and T5 in different genotypes under long day (LD), shift (Sh) and short day (SD). Black squares and lines indicate upregulated genes, gray squares and lines indicate downregulated genes.

Supplemental Figure S3

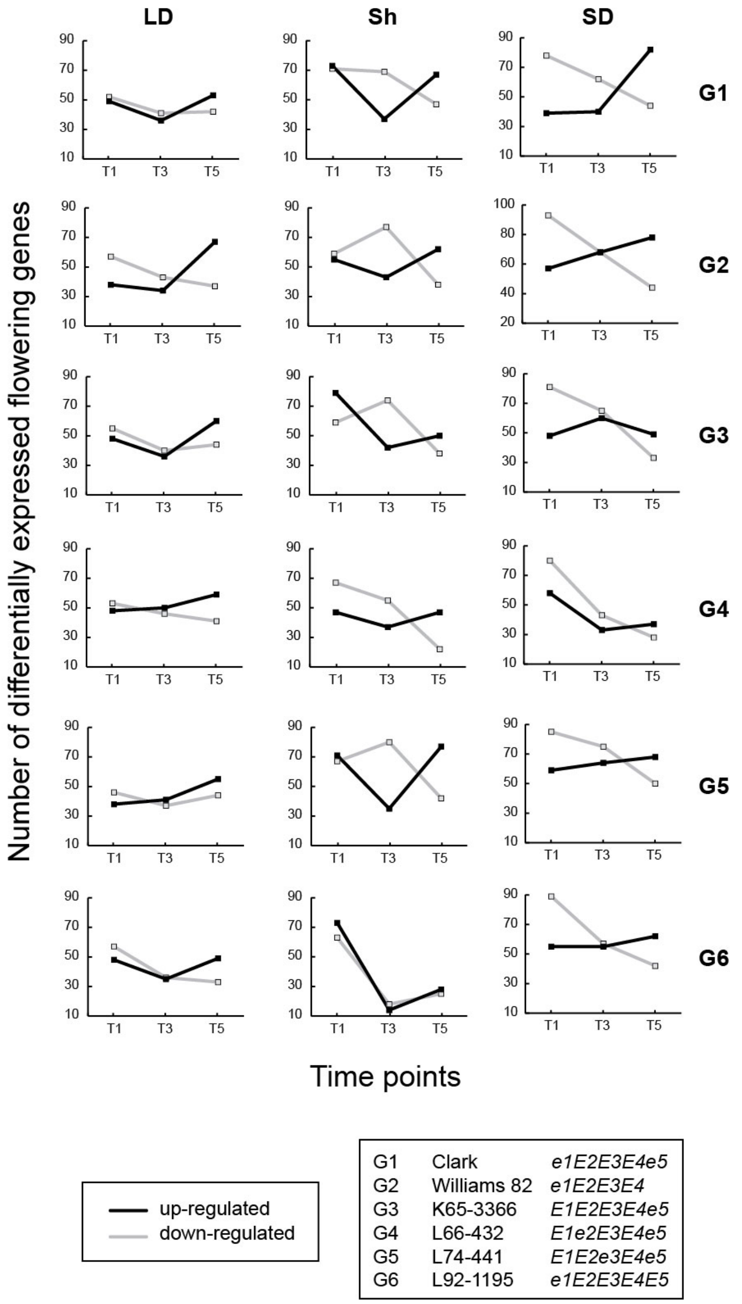

Supplemental Figure S3. Numbers of differentially expressed flowering genes at time points T1, T3 and T5 in different genotypes under long day (LD), shift (Sh) and short day (SD). Black squares and lines indicate upregulated genes, gray squares and lines indicate downregulated genes.

Supplemental Figure S4

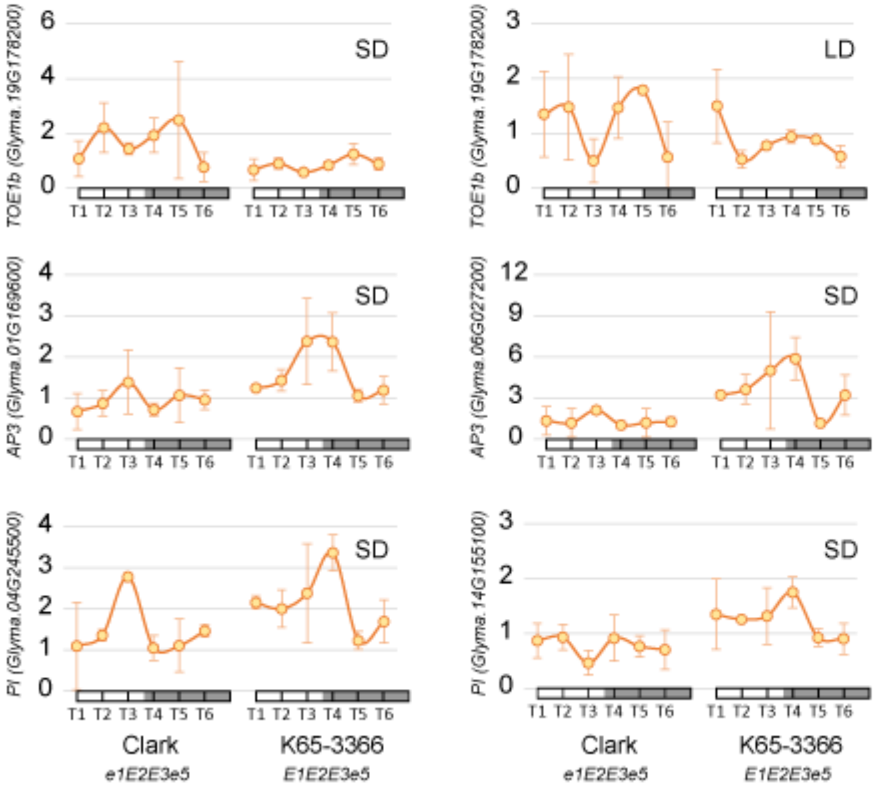

Supplemental Figure S4. Expression of *GmTOE1b* and soybean homologs of *AP3* and *PI* in Clark and the *E1* NIL under long day (LD) or short day (SD) by qRT-PCR. SD: 10-hour light (6:45–16:45), LD: 16-hour light (6:45–22:45). Time points T1–T6 represent 6:30, 10:30, 14:30, 18:30, 22:30 and 2:30. Error bars indicate standard deviation.

Supplemental Figure S5

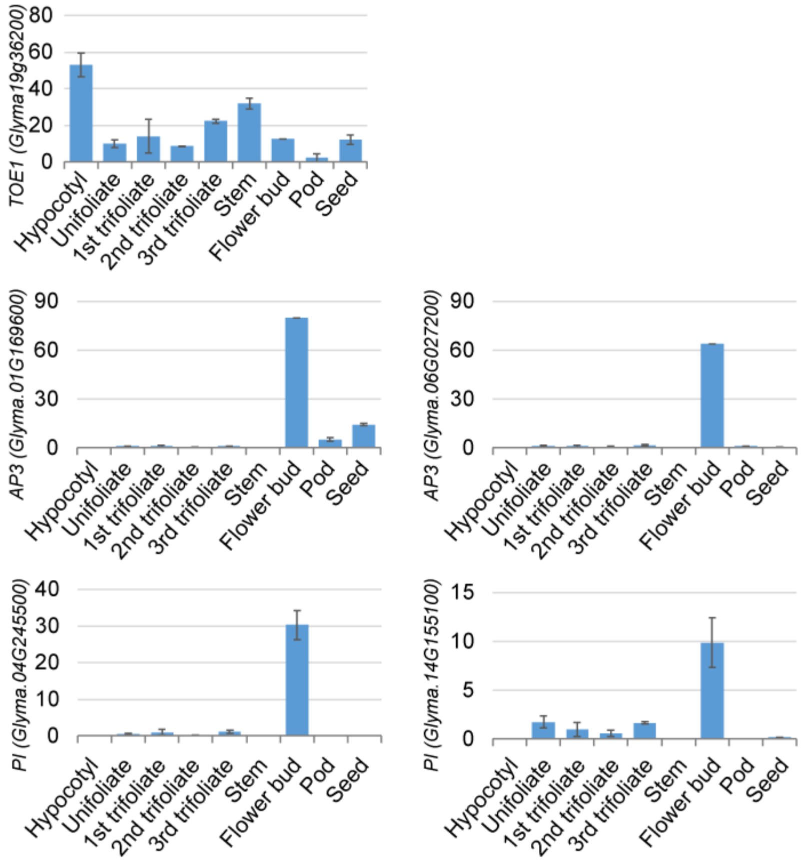

Supplemental Figure S5. Tissue-specific expression of *GmTOE1b* and *AP3* and *PI* homologs in Williams 82 grown under short day (10-hour light, 6:45–16:45) at 28°C by qRT-PCR. Hypocotyl, unifoliate, 1st trifoliate, 2nd trifoliate, 3rd trifoliate, stem, flower bud, pod and seed were sampled at 30 days after sowing at 13:00. Error bars indicate standard deviation.

Supplemental Figure S6

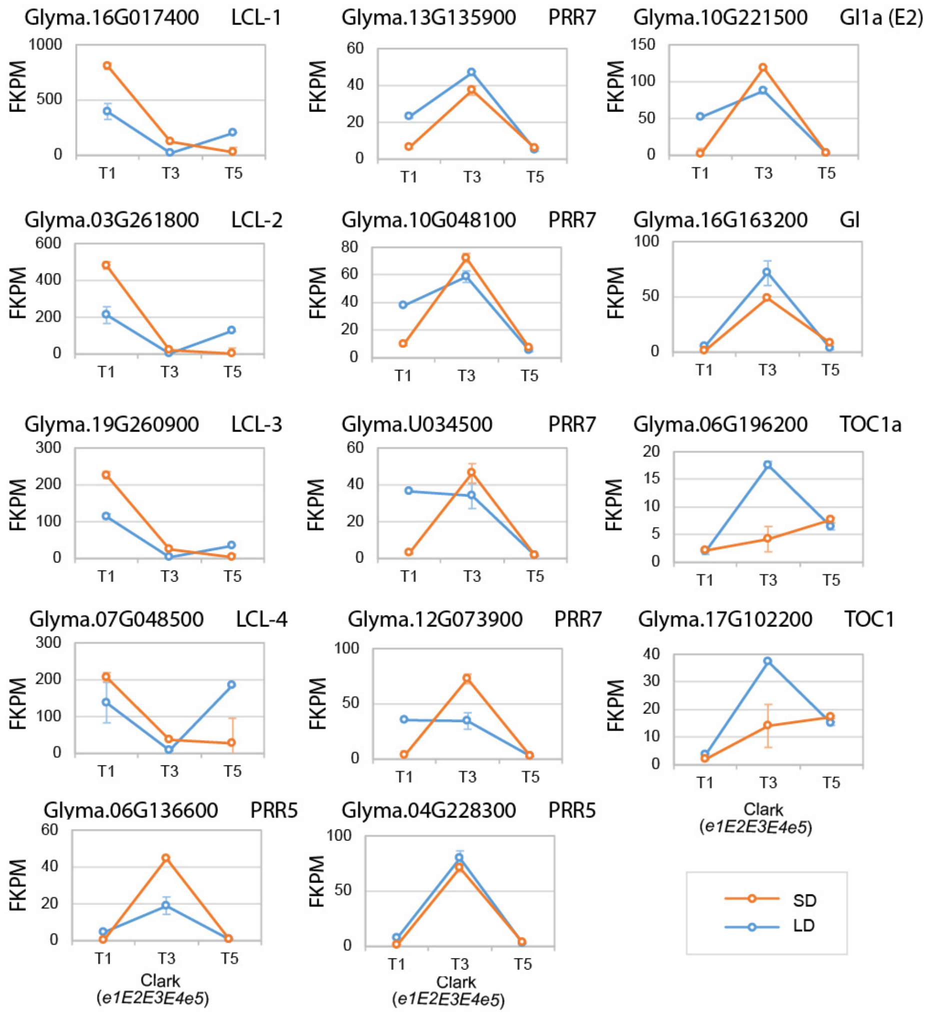

Supplemental Figure S6. Expression of soybean homologs of the circadian clock genes measured by RNA sequencing in Clark at three time points (T1: 6:30, T3: 14:30, T5: 22:30) under short day (SD: 10-hour light, 6:45–16:45) or long day (LD: 16-hour light, 6:45–22:45) three weeks after germination. FPKM values are displayed on the y-axes.

Supplemental Figure S7

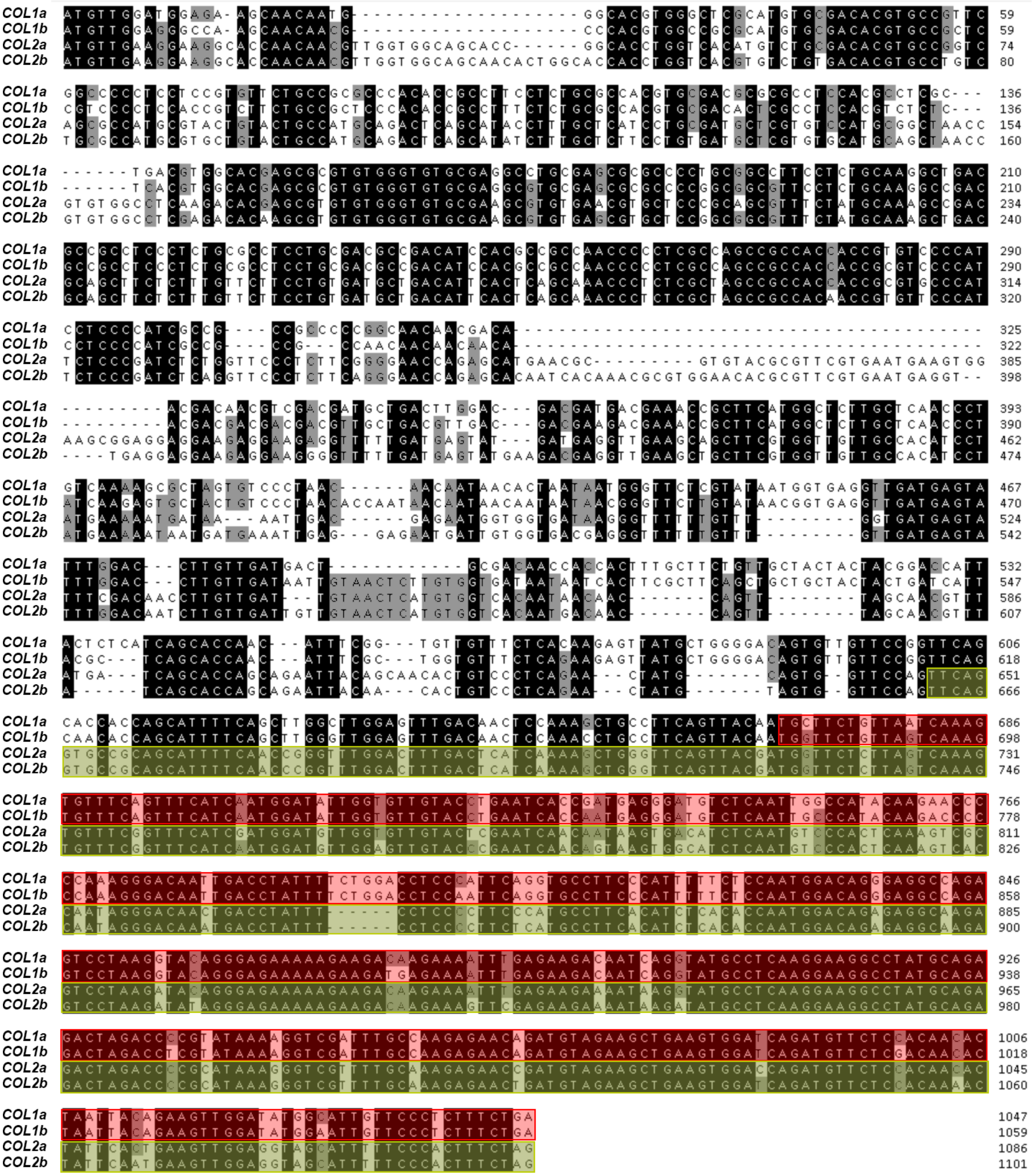

Supplemental Figure S7. Alignment of *GmCOL1a*, *GmCOL1b*, *GmCOL2a* and *GmCOL2b* cDNA sequences. The regions highlighted in red and yellow are the target regions of *COL1*-RNAi and *COL2*-RNAi, respectively. Sequences were retrieved from Phytozome. Dashes represent alignment indels. Black and gray shades indicate nucleotide positions shared by four and three *GmCOL* genes, respectively.
