# Supplementary material for: Transcriptome-Enabled Network Inference Revealed the GmCOL1 Feed-Forward Loop and Its Roles in Photoperiodic Flowering of Soybean
Source: Front Plant Sci. 2019 Nov 8;10:1221. doi: 10.3389/fpls.2019.01221 (PMC6856076; doi:10.3389/fpls.2019.01221)
Supplement: Supplementary file 3 [file DataSheet_2.pdf]

On validation of CausNet

In this document we describe an attempt to validate CausNet on *Arabidopsis* data. Such validation requires having data to input into the algorithm and a ground truth with which to compare the resulting signed network graph produced by the algorithm. As a ground truth network we take the network identified in [1]. This paper derives a differential equation model for the flowering network of *Arabidopsis* involving five genes, *FT*, *TFL1*, *FD*, *LFY*, and *AP1*. The expression level of *FT* is considered to be an input to the network, with a linearly increasing input function. The expression level of *AP1* is considered to be the output, with the times to cross certain thresholds determining numbers of rosette and cauline leaves. A differential equation model is proposed in [1] based on previous literature, and evidence for it is given in the paper. The model involves two complexes, the *FT*/*FD* complex and the *TFL1*/*FD* complex, as shown in Figure 1(A). The expression level of *FD* contributes positively to both complexes, but the complexes have opposite effects on expression levels of genes *LFY* and *AP1*. Thus, the graphical model of [1] does not map to a signed directed graph model. However, aside from the ambiguity in the sign of influence of *FD* on *AP1* and *LFY*, the influences of the genes on each other are clear and the resulting directed graph is shown in Figure 1(B).

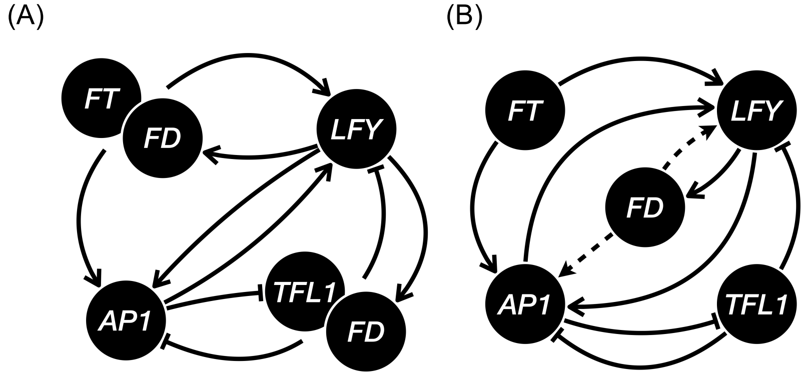

Figure 1: Core flowering networks of *Arabidopsis thaliana*. (A) The network of five gene hubs from Jaeger et al. with minor correction. (An edge from *FD* to *LFY* was confirmed to be incorrect with an author, and is replaced by the correct one from the *FT*/*FD* complex to *LFY*.) (B) Graph model with signed directed edges based on the flowering network of part (A). The signs of the two dashed edges could be positive or negative, depending on the relative abundance of *FT* (gives *FD* a positive influence on *AP1* and *LFY*) vs. *TFL1* (gives *FD* a negative influence on *AP1* and *LFY*), as seen in (A).

As for the data to input in the CausNet algorithm, we make use of the differential equation model, including the numerical values of the parameters, from [1]. These parameters were determined in [1] by fitting the model output to phenotype data (i.e., the number of leaves of two types). That is, we produced expression level data by numerically simulating the expression levels of the genes using a state of the art experimentally fit detailed model for the expression levels. Using the same initial conditions as in [1], Supplemental Figure 2, we found the differential equations reproduced the trajectories in that figure. To produce a variety of trajectories, simulating the effect of different conditions, we generated ten sets of trajectories by using randomly selected initial conditions, with the

five expression levels being independent and uniformly distributed over [0; 1]. Details of the code, including all parameter settings, can be found at https://github.com/Veggente/flowering-data-gen.

Two reconstructed networks produced by our software CausNet based on sparse linear regression and Granger causality are shown in Figure 2. The network shown on the left is produced using three time points, and ten sets of initial conditions for a total of thirty samples. The network shown on the right is produced using ten time points, ten sets of initial conditions for a total of 100 samples. The outputs bear a definite correlation to the ground truth represented in Figure 1(B). A similar approach could be undertaken using the experimentally fit model from [2] involving both the circadian clock network and flowering network of *Arabidopsis*. Additional research will be needed before algorithms can be shown to consistently and accurately reconstruct the true gene regulatory network for flowering of *Arabidopsis* based on limited amounts of expression level data.

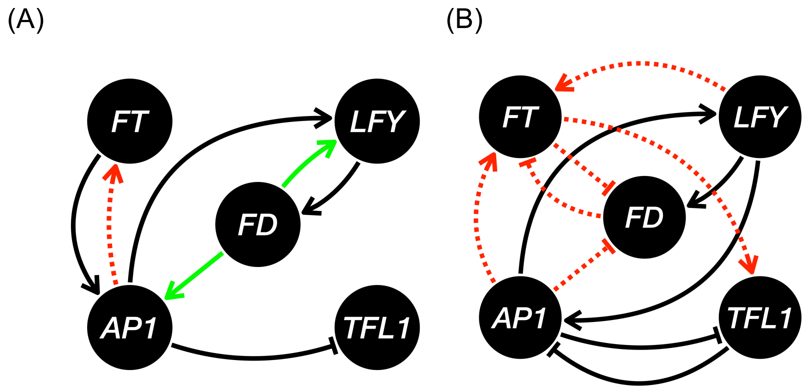

Figure 2: Network reconstruction using CausNet. (A) Reconstruction with three time points. (B) Reconstruction with ten time points. The solid black edges are true edges in Figure 1(B). The red dashed edges are false discoveries in Figure 1(B). The green edges can be positive or negative in Figure 1(B).

References

[1] K.E. Jaeger, N. Pullen, S. Lamzin, R.J. Morris, and P.A. Wigge. Interlocking feedback loops govern the dynamic behavior of the floral transition in *Arabidopsis*. *The Plant Cell*, 25(3):820–833, 2013.

[2] Daniel D Seaton, Robert W Smith, Young Hun Song, Dana R MacGregor, Kelly Stewart, Gavin Steel, Julia Foreman, Steven Penfield, Takato Imaizumi, Andrew J Millar, and Karen J Halliday. Linked circadian outputs control elongation growth and flowering in response to photoperiod and temperature. *Molecular Systems Biology*, 11(1), 2015. ISSN 1744-4292. doi: 10.15252/msb.20145766. URL http://msb.embopress.org/content/11/1/776.
